# Inseparability of Go and Stop in Inhibitory Control: Go Stimulus Discriminability Affects Stopping Behavior

**DOI:** 10.3389/fnins.2016.00054

**Published:** 2016-03-22

**Authors:** Ning Ma, Angela J. Yu

**Affiliations:** ^1^Department of Electrical and Computer Engineering, University of California, San DiegoLa Jolla, CA, USA; ^2^Department of Cognitive Science, University of California, San DiegoLa Jolla, CA, USA

**Keywords:** decision making, Bayesian modeling, perceptual uncertainty, inhibitory control, stop signal task

## Abstract

Inhibitory control, the ability to stop or modify preplanned actions under changing task conditions, is an important component of cognitive functions. Two lines of models of inhibitory control have previously been proposed for human response in the classical stop-signal task, in which subjects must inhibit a default *go* response upon presentation of an infrequent *stop* signal: (1) the *race model*, which posits two independent *go* and *stop* processes that race to determine the behavioral outcome, go or stop; and (2) an *optimal decision-making model*, which posits that observers decides whether and when to go based on continually (Bayesian) updated information about both the go and stop stimuli. In this work, we probe the relationship between *go* and *stop* processing by explicitly manipulating the discrimination difficulty of the go stimulus. While the race model assumes the go and stop processes are independent, and therefore go stimulus discriminability should not affect the stop stimulus processing, we simulate the optimal model to show that it predicts harder go discrimination should result in longer go reaction time (RT), lower stop error rate, as well as faster stop-signal RT. We then present novel behavioral data that validate these model predictions. The results thus favor a fundamentally inseparable account of go and stop processing, in a manner consistent with the optimal model, and contradicting the independence assumption of the race model. More broadly, our findings contribute to the growing evidence that the computations underlying inhibitory control are systematically modulated by cognitive influences in a Bayes-optimal manner, thus opening new avenues for interpreting neural responses underlying inhibitory control.

## 1. Introduction

**Figure 1:**
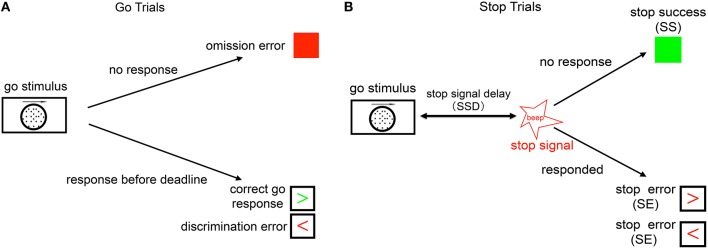
**Schematic illustration of the stop signal task. (A)** Go trials: on go trials, subject is supposed to make a response to a default go response by pressing the left or right button, based on the coherent motion direction of random dots. The go reaction time (Go RT) is defined as the time the subject takes to respond from the onset of go stimulus. The subject makes a discrimination error if he/she chooses the wrong direction (wrong key), and an omission error if no response is recorded within the response deadline (1100 ms). **(B)** Stop trials: on a small fraction of trials, a stop signal appears after the go stimulus and instructs the subject to withhold the go response. The time delay between the go stimulus onset and the stop signal onset is called the stop-signal delay (SSD). If the subject makes a go response in a stop trial, this trial is considered a stop error (SE) trial, otherwise it is considered a stop success (SS) trial.

The ability to cancel or modify planned actions according to changing task conditions is known as *inhibitory control*, and thought to be an important aspect of human cognitive function. Inhibitory control has been studied extensively using the stop-signal (Logan and Cowan, 1984), in which subjects typically discriminate a *go* stimulus on each trial, but occasionally encounter a *stop* signal following the go stimulus, which instructs the subject to withhold the go response (see Figure 1). Two major class of models have been proposed to account for the underlying computational and neural processes in the stop-signal task. The first is the classical race model and its variants (Logan and Cowan, 1984; Boucher et al., 2007), which posit a race between two independent go and stop processes. The model assumes essentially immutable, though noisy, termination times for the go and stop processes, whereby the average stop process delay, known as the stop-signal reaction time (SSRT), is generally thought to be a measure of an individual's inhibitory capacity. Correspondingly, SSRT has been measured as longer in populations with presumed inhibitory deficits (Nigg et al., 2006; Alderson et al., 2007; Menzies et al., 2007), and neural activities in certain primate brain regions (e.g., frontal eye field and superior colliculus) have been interpreted to reflect components of the race model(Hanes et al., 1998; Pare and Hanes, 2003). However, problematic for the simple race model, various cognitive contextual factors have been shown to systematically modulate stopping behavior, such as the reward structure of the task (Leotti and Wager, 2009) and the statistical frequency of stop signals (Emeric et al., 2007). In response to these and other observed cognitive influences, we previously proposed an alternative model of inhibitory control, a Bayes-optimal decision-making model positing that subjects choose when and whether to initiate a go response according to continually (Bayesian) updated sensory beliefs about both the go and stop stimuli, and relative to a behavioral objective function that penalize go and stop errors as well as response delay. As we previously showed, this optimal model can capture cognitives influences on stopping behavior as a function of sensory statistics at multiple timescales (Ide et al., 2013; Ma and Yu, 2015a,b) and the reward structure of the task (Shenoy and Yu, 2011).

In this work, we explore a particular type of interaction between go and stop processing in the stop-signal task. Specifically, we consider the computational and behavioral consequences of manipulating the go stimulus discrimination difficulty. We will use simulations of the optimal model to demonstrate that, as the go stimulus becomes noisier (harder to discriminate), the go reaction time (RT) should get longer and consequently the rate of stop errors to drop, as subjects have a greater opportunity to detect the late-appearing stop signals before initiating the go response; perhaps less obviously, SSRT becomes longer, but does not do so sufficiently to counter the longer go RT, as over all stop error rate still decreases. We will then present novel experimental data from human subjects (*n* = 20) performing a stop-signal task, in which the go task is to discriminate a random-dot coherent motion stimulus (Britten et al., 1992), and the stimulus difficulty (coherence) is varied across blocks. The key question is whether SSRT co-varies with stimulus coherence, as predicted by the optimal model, or whether stopping behavior is independent of go discriminability, as assumed by the race model. More broadly, we will examine if the spectrum of behavioral measures—SSRT, go RT, and stop error rate—changes systematically with respect to go stimulus coherence as predicted by the optimal model, as that would yield further evidence that human inhibitory control is under sophisticated, context-sensitive, and statistically optimal cognitive control.

In the following, we first describe the experimental procedure (Section 2.1), then model details (Section 2.2), followed by model simulation results and behavioral data. We conclude with some discussions and thoughts on related work and future directions (Section 4).

## 2. Materials and methods

### 2.1. Experiment

The stop signal task consists of a two alternative forced-choice (2AFC) perceptual discrimination task, interspersed with an occasional stop signal. Figure 1 schematically illustrates our version of the stop-signal task: subject responds to a default go stimulus on each trial (go trial), unless instructed to withhold the response by an infrequent auditory stop signal (stop trial). The go task is either a random-dot coherent motion task (8, 15, or 85% coherence), or a more classical square vs. circle discrimination task. On a small fraction (25%) of trials, an additional auditory *stop* signal (a beep) occurs at some time after the go stimulus onset (known as the stop-signal delay, or SSD), which instructs the subject to withhold the *go* response. The SSD is randomly and uniformly sampled on each trial from 100, 200, 300, 400, 500, and 600 ms.

We say that the subject makes a discrimination error when he/she incorrectly responds to the stimulus on go trials, i.e., choosing the opposite motion direction or incorrect shape. The subject makes an omission error if he/she fails to make a go response prior to the response deadline on a go trial, set to be 1100 ms in the experiment. The trials having stop signal are called stop trials; trials without stop signal are go trials. When the subject withholds the response until the response deadline on a stop trial, the trial is considered a stop success (SS) trial; otherwise, it is considered a stop error (SE) trial. Each trial is terminated when the subject makes a response, or at the response deadline itself if no response has been recorded. To incentivize the subjects to be engaged in the task, and to help standardize the relative costs of the different kind of errors across individuals, subjects are compensated proportional to points they earn in the task, whereby they lose 50 points for a go discrimination or omission error, 50 points for a stop error, and 3 points for each 100ms of response delay (so maximally 33 points for a trial that terminates with no response, and less if the subject makes a response prior to the response deadline).

Twenty subjects (13 females) participated in the experiment. Each subject performed 12 blocks, 3 block for each stimulus type, with each block containing 75 trials. Two days before the main experiment session, subjects participated in a training session, which contained only the 2AFC discrimination and no stop trials. In the training session, there were 10 blocks, 3 blocks for each random dot stimulus coherence and one block for shape discrimination. Subjects were given the same maximal amount of time to respond on the training session trials (1100 ms) as in the main experiment. The purpose of the training session is to allow subjects to familiarize themselves with the task and to achieve stable perceptual discrimination performance. Only data from the main experimental session are analyzed and presented here.

This experimental protocol was approved by the University of California San Diego Human Subjects Review Board, and all subjects gave written informed consent.

### 2.2. Model

#### 2.2.1. The race model

**Figure 2:**
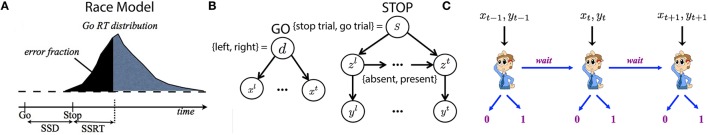
**Model illustration. (A)** Classical race model for behavior in the stop-signal task. The behavioral outcome (go or stop) is determined by a race between a go and a stop process. The go reaction time has a broad distribution due to noise. The stop process has an average delay known as the *stop signal reaction time* (SSRT). The stop error rate is the cumulative density of Go RT at SSD + SSRT. The SSRT is thus estimated from data as the difference between the median go RT and the SSD at which 50% stop error is achieved, as SSD+SSRT = median(go RT) implies 50% of stop trials will end in error (the rest in success). **(B)** Bayesian generative model of iid sampled sensory observations (*x*^1^, …, *x*^*t*^, …) conditioned on Go stimulus identity (*d* = 0 of left, *d* = 1 for right), and an independent stream of observations (*y*^1^, …, *y*^*t*^, …) conditioned on the presence (*z*^*t*^ = 1) or absence (*z*^*t*^ = 0) of the Stop signal, which has a geometrically distributed onset time when it is a stop trial *s* = 1 and never appears on a go trial (*s* = 0). **(C)** The decision of whether/when to Go, and which Go response to select, are modeled as sequential decision-making, where the subject chooses at each moment whether to select a Go response, or to wait at least one more time point.

The classical race model for studying inhibitory control is shown in Figure 2A. The subject makes a stop error when the *go* response is finished processing before the stop process. The race model also defines a subject-specific *stop-signal reaction time* (SSRT), which is a measure of the average amount of time the subject requires to process the stop signal and cancel the go response (in practice, it is often calculated as the difference between the median go RT and the SSD specific to each subject for achieving 50% accuracy on stop trials). SSRT is thought to index an individual's stopping ability, and has been observed to be longer in various psychiatric conditions thought to involve inhibitory deficits (e.g., substance abuse Nigg et al., 2006, attention-deficit hyperactivity disorder Alderson et al., 2007, schizophrenia Badcock et al., 2002, obsessive-compulsive disorder Menzies et al., 2007).

#### 2.2.2. Optimal inhibitory control model

Recently, we proposed a normative Bayesian Markov decision process (MDP) model for the stop-signal task (Shenoy and Yu, 2011), which assumes that the subjects maintain continually evolving Bayes-optimal beliefs about the sensory environment (see Figure 2B), and that they make moment-by-moment decisions between *go* and *wait* by mapping the current belief state into the action space, in a manner consistent with optimizing a global objective function an objective function (Figure 2C). The behavioral objective function is assumed to take into account the costs associated with go errors, stop errors, and response delay.

Figure 2B illustrates the Bayesian generative model for how iid noisy sensory data are assumed to be generated by the (true) hidden stimulus states. The two hidden variables *d* and *s* correspond respectively to the identity of the go stimulus, *d*∈{0, 1} (0 for left, 1 for right), and whether or not this trial is a stop trial, *s*∈{0, 1}. *P*(*s* = 1) and *P*(*d* = 1) are the prior probability of a stop trial and one of two go alternatives, respectively. Conditioned on the go stimulus identity *d*, a sequence of iid sensory inputs, corresponding to noisy information about the go stimulus, are assumed to be generated on each trial, *x*^1^, …, *x*^*t*^, …, where *t* indexes time steps *within a trial*. The likelihood functions of *d* generating the sensory inputs are $f_{0}\left(x^{t}\right)=p\left(x^{t}|d=0\right)$ and $f_{1}\left(x^{t}\right)=p\left(x^{t}|d=1\right)$, which are assumed to be Bernoulli distribution with respective rate parameters *q*_*d*_ and 1−*q*_*d*_. The parameter *q*_*d*_ specifies stimulus signal-to-noise ratio, thus reflecting the go stimulus difficulty. The discrimination task becomes harder (lower coherence) when *q*_*d*_ is closer to 0.5 and easier (high coherence) when *q*_*d*_ is closer to 1 or 0. The dynamic variable *z*^*t*^ denotes the presence/absence of the stop signal. *z*^1^ = … = *z*^θ−1^ = 0 and *z*^θ^ = *z*^θ+1^ = … = 1 if a stop signal appears at time θ, where θ represents stop signal delay SSD. For simplicity, we assume that θ, also known as the stop-signal delay (SSD), follows a geometric distribution: *P*(θ = *t*|*s* = 1) = *q*(1−*q*)^*t*−1^. The expected value of θ is 1∕*q*, which is the expected SSD, *E*[*SSD*], within a trial. Conditioned on *z*^*t*^, each observation *y*^*t*^ is independently generated and corresponds to one unit of noisy information about the stop signal. For simplicity, we assume the likelihood, $p\left(y^{t}|z^{t}=0\right)=g_{0}\left(y^{t}\right)$ and $p\left(y^{t}|z^{t}=1\right)=g_{1}\left(y^{t}\right)$, are Bernoulli distributions with rate parameters *q*_*s*_ and 1−*q*_*s*_, respectively.

In the statistically optimal recognition model, Bayes' Rule is applied in the usual iterative manner to compute the iterative posterior probability associated with go stimulus identity, $p_{d}^{t}:=P\left(d=1|{\text{x}}^{t}\right)$, the presence of the stop signal, $p_{z}^{t}:=P\left(\theta \leq t|{\text{y}}^{t}\right)$, and whether the current trial is a stop trial, $p_{s}^{t}:=P\left(s=1|{\text{y}}^{t}\right)$, where **x**^*t*^ = {*x*^1^, *x*^2^, …, *x*^*t*^} and **y**^*t*^ = {*y*^1^, *y*^2^, …, *y*^*t*^} denote all the data observed so far. The *belief state* at time *t* is defined to be the vector ${\text{b}}^{t}=\left(p_{d}^{t},p_{s}^{t}\right)$, which can be iteratively computed from time step to time step via Bayes' Rule, by inverting the generative model (Figure 2B) as follows,

$$\begin{array}{l} p_{d}^{t}=\frac{p_{d}^{t-1}f_{1}(x^{t})}{p_{d}^{t-1}f_{1}(x^{t})+(1-p_{d}^{t-1})f_{0}(x^{t})}. \end{array}$$

To infer the stop signal, we first update iteratively by

$$\begin{array}{l} p_{z}^{t}=\frac{g_{1}(y^{t})(p_{z}^{t-1}+(1-p_{z}^{t-1})h(t))}{g_{1}(y^{t})(p_{z}^{t-1}+(1-p_{z}^{t-1})h(t))+g_{0}(y^{t})(1-p_{z}^{t-1})(1-h(t))} \end{array}$$

where h(t) is the posterior probability that the stop-signal will appear in the next time step given that it has not appeared yet,

$$\begin{array}{l} h(t)=\frac{rP(\theta =t|s=1)}{rP(\theta \text{ ​}>\text{ ​}t-1|s=1)+(1-r)}=\frac{rq{\left(1-q\right)}^{t-1}}{r{\left(1-q\right)}^{t-1}+(1-r)} \end{array}$$

where *r* = *P*(*s* = 1) is the prior probability of a stop trial. The posterior probability that the current trial is a stop trial can be computed as

$$\begin{array}{l} p_{s}^{t}=P(s=1|y^{t})=p_{z}^{t}+(1-p_{z}^{t})P(s=1|\theta \text{​ }>\text{ ​}t,y^{t}) \end{array}$$

where *P*(*s* = 1|θ > *t*, **y**^*t*^) is independent from the past observations **y**^*t*^

$$\begin{array}{l} P(s=1|\theta \text{ ​}>\text{ ​}t,y^{t})=\\ \;\frac{P(\theta \text{ ​}>\text{ ​}t|s=1)P(s=1)}{P(\theta \text{ ​}>\text{ ​}t|s=1)P(s=1)+P(\theta \text{ ​}>\text{ ​}t|s=0)P(s=0)}\\ \;=\frac{{\left(1-q\right)}^{t}r}{r{\left(1-q\right)}^{t}+(1-r)}. \end{array}$$

Figure 2C illustrates the sequential decision-making process that determines how an observer chooses whether/when to Go, and which Go response to select. The Markov decision process is optimized with respect to the Bayesian belief state and a behaviorally defined cost function that captures the cost and penalty structure of SST, based on which the observer decides at each moment in time whether to Go (and if so, which Go response) or Wait at least one more time step.

On each trial, if the Go action is taken by the response deadline *D*, it is recorded as a Go response (correct on Go trials, error on Stop trials); otherwise the trial is terminated by the response deadline and a Stop response is recorded (omission error on Go trials, correct on Stop trials). Let τ denote the trial termination time, so that τ = *D* if no response is made before the deadline *D*, and τ < *D* if a Go action is chosen. δ∈{0, 1} represents the possible binary Go choices produced by making a Go response. We assume there is a cost *c* incurred per unit time in response delay (corresponding to time-dependent costs, such as time, effort, opportunity, or attention), a stop error penalty of *c*_*s*_ for responding on a Stop trial, and a unit cost for making a discrimination error or ommission error on a Go trial—since the cost function is invariant with respect to scaling, we normalize all cost parameters relative to the Go error cost without loss of generality. Thus, the cost function is:

$$\begin{array}{l} l(\tau ,\delta ;d,s,\theta ,D)=c\tau +c_{s}1_{\left\{\tau <D,s=1\right\}}+1_{\left\{\tau <D,\delta \neq d,s=0\right\}}\\ \;+\;1_{\left\{\tau =D,s=0\right\}} \end{array}$$

where **1** denotes the indicator function, which evaluates to 1 when the condition specified in the curly brackets are met, and to 0 otherwise.

The optimal decision policy minimizes the expected (average) loss, *L*_π_ = 𝔼[*l*(τ, δ; *d, s*, θ, *D*)],

$$\begin{array}{l} L_{\pi }=c𝔼\left[\tau \right]+c_{s}rP\left(\tau <D|s=1\right)\\ +\left(1-r\right)P\left(\tau <D,\delta \neq d|s=0\right)+\left(1-r\right)P\left(\tau =D|s=0\right) \end{array}$$

which is an expectation taken over hidden variables, observations, and actions, and is generally computationally intractable to minimize directly. Fortunately, by formulating the problem as a belief state Markov decision process, we can use standard dynamic programming (Bellman, 1952) to compute the optimal policy and action via a recursive relationship between the value function and the Q-factors. The value function *V*^*t*^(**b**^*t*^) denotes the expected cost of taking the optimal policy henceforth when starting out in the belief state **b**^*t*^. The Q-factors, $Q_{g}^{t}\left({\text{b}}^{t}\right)$ and $Q_{g}^{w}\left({\text{b}}^{t}\right)$, denote the minimal costs associated with taking the action Go or Wait, respectively, when starting out with the belief state **b**^*t*^, and subsequently adopting the optimal policy. The Bellman dynamic programming principle, applied to our problem, implies:

$$\begin{array}{l} Q_{g}^{t}(b^{t})=ct+c_{s}p_{s}^{t}+(1-p_{s}^{t})\text{min}(p_{d}^{t},1-p_{d}^{t})\\ Q_{w}^{t}(b^{t})=1_{\left\{D>t+1\right\}}𝔼\text{[}V^{t+1}(b^{t+1})|b^{t}{\text{]}}_{b^{t+1}}\\ \;+\;1_{\left\{D=t+1\right\}}(c(t+1)+1-p_{s}^{t})\\ V^{t}(b^{t})=\text{min}(Q_{g}^{t},Q_{w}^{t}) \end{array}$$

whereby the optimal policy in state **b**^*t*^ is to choose between Go and Wait depending on which one has the smaller expected cost. Note that a Go response terminates the current trial, while a Wait response lengthens the current trial by at least one more time step, and repeatedly choosing Wait until the response deadline constitutes a Stop response. Since the observer can no longer update the belief state or take any action at the deadline, the value function at *t* = *D* can be computed explicitly, without recursion, as $V^{t}\left({\text{b}}^{D}\right)=cD+\left(1-P_{s}^{D}\right)$. Bellman's equation then allows us compute the value functions and Q factors exactly, backward in time from *t* = *D* −1 to *t* = 1.

We note that the decision problem can also be formalized as a mathematically equivalent partially observable Markov decision process (POMDP), whereby the hidden state is the stimulus state (*d, s*), the observations are iid noisy samples conditioned on that hidden state, and actions are chosen (Go or Wait) based on all previous observations as well as any prior beliefs about the hidden state. However, it is a rather trivial sort of POMDP, as not only do the actions not affect the hidden state, but the hidden state does not have any dynamics at all. Instead, we chose to formulate the problem as a (belief) Markov Decision Process, whereby the hidden state at time *t* is the posterior distribution over the stimuli at time *t* (the initial state is just the the prior distribution), and its (non-trivial) evolution over time is governed exactly by Bayes' Rule, applied to the previous posterior state and the new observation, and is completely observed. The only caveat is that the belief state is a continuous variable, and thus in order to apply Bellman's dynamic programming equation, we have to discretize the belief state. In the simulations, we discretize the belief state space, $\left(p_{d}^{t},p_{s}^{t}\right)$, into 200 × 200 bins.

Bayes rule implies the belief state **b**^*t*+1^ is a deterministic function of **b**^*t*^ and the observations. Thus, given *V*^*t*+1^, we can compute 𝔼[*V*^*t*+1^] by averaging over all possible next observations *x*^*t*+1^,*y*^*t*+1^.

$$\begin{array}{l} 𝔼\left[V^{t+1}\left({\text{b}}^{t+1}\right)|{\text{b}}^{t}\right]=\underset{x^{t+1},y^{t+1}}{\sum }p\left(x^{t+1},y^{t+1}|{\text{b}}^{t}\right)\\ \;\times V^{t+1}\left({\text{b}}^{t+1}\left({\text{b}}^{t},x^{t+1},y^{t+1}\right)\right)\\ p\left(x^{t+1},y^{t+1}|{\text{b}}^{t}\right)=p\left(x^{t+1}|p_{d}^{t}\right)p\left(y^{t+1}|p_{s}^{t}\right)\\ p\left(x^{t+1}|p_{d}^{t}\right)=p_{d}^{t}f_{1}\left(x^{t+1}\right)+\left(1-p_{d}^{t}\right)f_{0}\left(x^{t+1}\right)\\ p\left(y^{t+1}|p_{s}^{t}\right)=\left(p_{z}^{t}+\left(1-p_{z}^{t}\right)h\left(t+1\right)\right)g_{1}\left(y^{t+1}\right)\\ +\;\left(1-p_{z}^{t}\right)\left(1-h\left(t+1\right)\right)g_{0}\left(y^{t+1}\right) \end{array}$$

**Figure 3:**
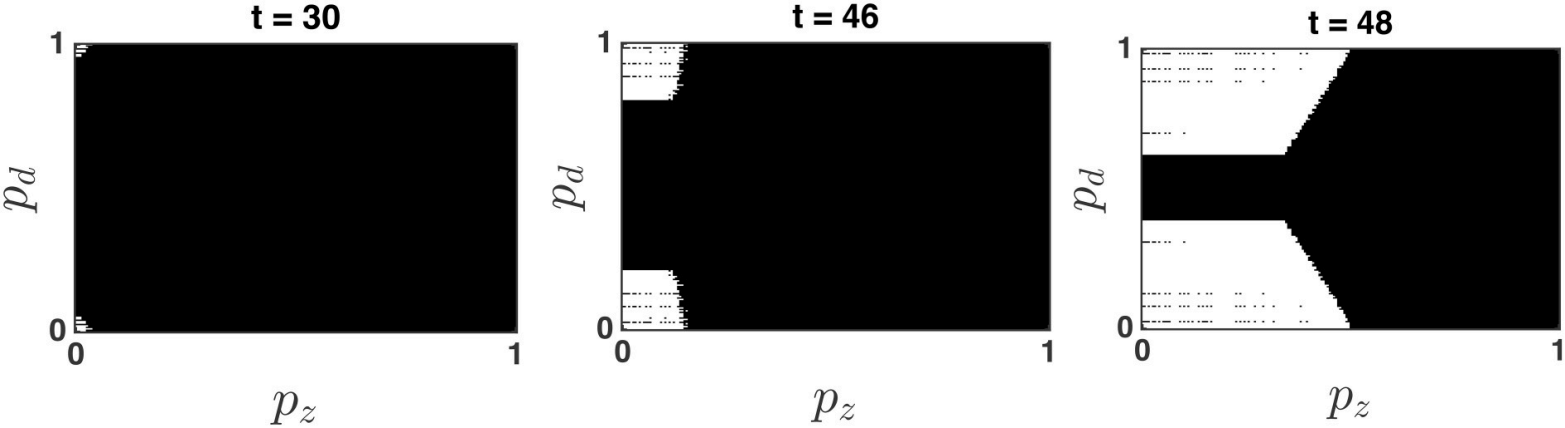
**Dynamic evolution of the optimal policy map**. The white and black areas denote the *Go* and *Wait* regions, respectively. Note that *p*_*z*_ denotes the probability that the stop signal has already occurred at time *t*, and is monotonically related to *p*_*s*_, the probability that the current trial is a stop trial. The Bayesian update algorithm produces a belief state (*p*_*d*_, *p*_*z*_) at every time point *t* based on prior belief and all sensory data **x**_*t*_, **y**_*t*_ observed until time *t*. If the belief state falls into a Go region, a Go response is produced and the trial is terminated; otherwise, at least one more observation is obtained, and the location of the new Bayesian-updated belief state is compared to next time point's optimal policy map. When/whether the belief state falls into a Go region determines when/whether the subject produces a response on that trial. The simulation shows that the Go regions expand over time, as the response deadline looms closer.

The optimal decision policy partitions the belief state into Go and Stop regions, such that the optimal decision is to go (and terminate the trial) if the belief state at time *t*, (*p*_*d*_, *p*_*z*_), falls into a Go region (where *Q*_*g*_ < *Q*_*w*_), and the optimal decision is to wait (at least one more time point, but with the possibility of going later before the deadline) if the belief state falls into a Wait region (where *Q*_*w*_ < *Q*_*g*_). Figure 3 shows that there are typically two symmetric Go regions, where *p*_*s*_ is relatively small, and *p*_*d*_ is close to 0 or 1 (i.e., the probability of a stop trial is small and the confidence about go stimulus identity, left or right, is high), and a large central *Wait* region, where the value of *p*_*d*_ is close to 0.5 (go stimulus identity highly uncertain) or the value of *p*_*s*_ is large (probability of stop trial high). This topology makes intuitive sense. Figure 3 also shows that the optimal decision policy is time-dependent, such that the Go regions grow over time. This is primarily due to the time pressure of the impending response deadline (Frazier and Yu, 2008).

## 3. Results

### 3.1. Model predictions

Classical behavioral results in the stop signal task, such as increases in stop error rate as a function of SSD and the generally faster SE RT compared to go RT, have been shown to be natural consequences of such a rational decision-making process (Shenoy and Yu, 2011), although these effects are also captured by the race model (Logan and Cowan, 1984). However, being a *computational* model in Marr's framework of levels of analysis (Marr, 1982), the optimal model is not only a model of the brain processes but also of the computational *task* the brain must solve—as such, it can also make *normative predictions* about how experimental manipulations of different task parameters should affect stopping behavior, since the experimental parameters are naturally represented as parameters of the Bayesian general model or of the objective function. In contrast, the race model cannot make such predictions, since it has no means of representing properties of the task itself.

**Figure 4:**
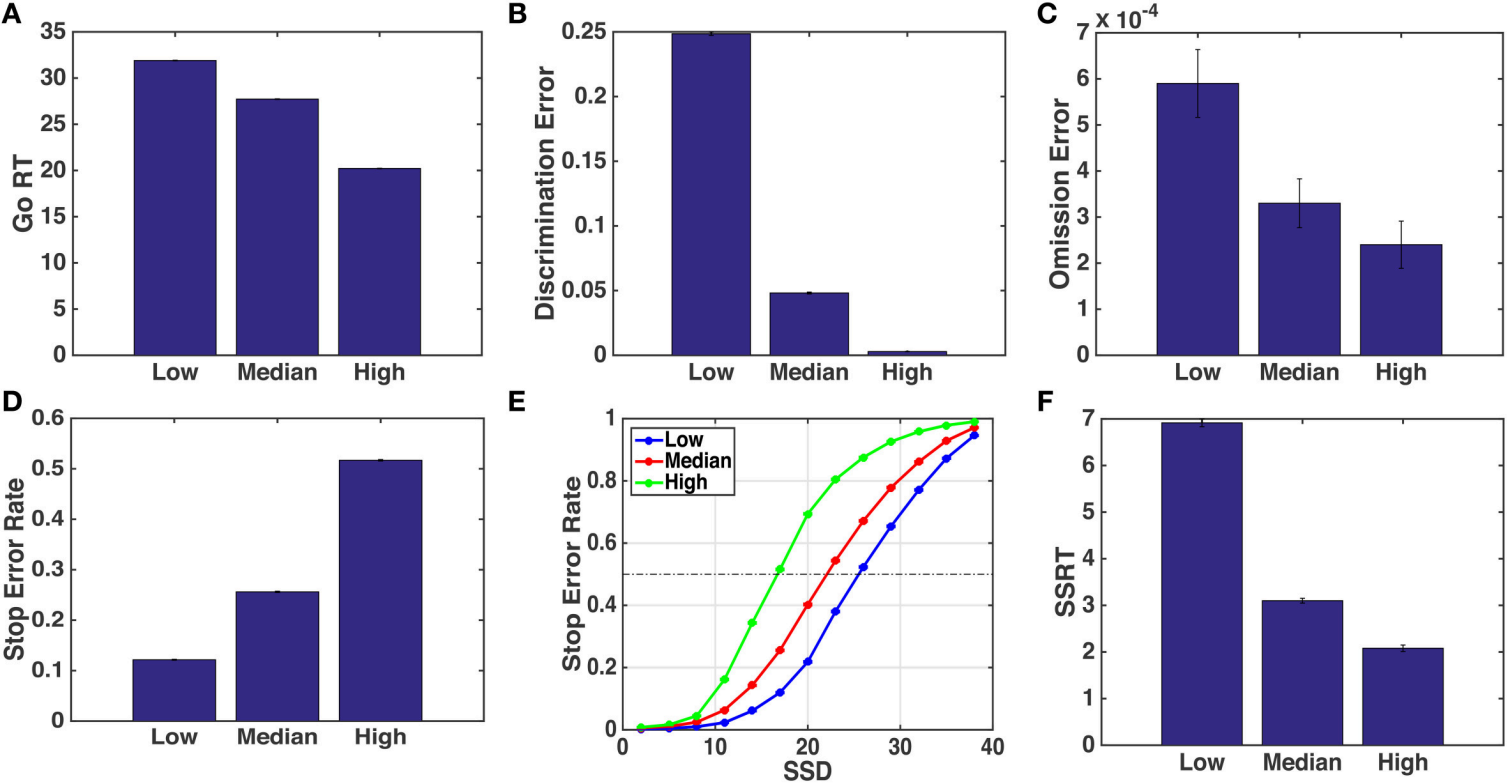
**Simulated behavioral predictions by the optimal decision-making model**. **(A)** Simulated mean Go RT decreases with higher go stimulus coherence (easier discrimination). *Low, median*, and *high* stimulus coherence are parameterized by different values of *q*_*d*_: 0.55, 0.62, and 0.70, respectively. **(B)** Discrimination error rate decreases as coherence increases. **(C)** Omission error rate decreases with *q*_*d*_. **(D)** Stop error rate increases with the *q*_*d*_. **(E)** Average stop error rate as a function of SSD (known as the *inhibition function*) for different stimulus conditions. **(F)** SSRT decreases with *q*_*d*_. Each data point is averaged over 100 simulated subjects, each performing 1000 go or stop trials. Error bars indicate standard error of the mean (sem); sem is extremely small and almost invisible for all but the omission error simulation data. Other parameters used in the model are adapted from Shenoy and Yu (2011): *r* = 0.25, *q*_*s*_ = 0.72, *D* = 50, *c*_*s*_ = 0.4, *c* = 0.002.

Here, we specifically focus on the behavioral consequences of changing go stimulus discrimination difficulty. The race model does not represent stimulus difficulty explicitly and thus would not make any obvious predictions about behavioral consequences; moreover, since the go and stop processes are assumed to be independent, the race model would certainly not predict properties of the stop process, such as the SSRT, to change with go stimulus difficulty. On the other hand, in the optimal model, changes in the go stimulus difficulty would change the evolution of the sensory belief state (both via the empirical statistics and the Bayesian update rule, since we assume subjects to have the correct generative model), as well as the decision policy (the time-dependent mapping between the belief state and the action set), whose computation of $Q_{w}^{t}$ involves an expectation over future belief state, which depends on the assumed likelihood function. Intuitively, we would expect a noisier go stimulus to slow down the general drift of the go stimulus posterior belief $p_{d}^{t}$ toward 0 or 1 (depending on which true stimulus was shown), and hit the “Go region” later on a go trial, or avoid hitting it altogether on a stop trial (Figure 3). Figure 4 show the simulated model predictions for the various behavioral measures as a consequence of changing go stimulus discrimination difficulty, parameterized by *q*_*d*_ in the model. We model *q*_*d*_ as monotonically decreasing (toward 0.5, which corresponds to a stimulus containing pure noise) for decreasing stimulus coherence. The exact values chosen for *q*_*d*_ in the simulations are as specified in Figure 4 caption. We find that the qualitative, monotonic relationships between the various predicted behavioral measures and the go stimulus coherence hold for a large range of *q*_*d*_ values chosen, as long as lower coherence corresponds to smaller *q*_*d*_. As shown in Figures 4A–C, we expect Go RT to decrease, and Go discrimination and omission errors to decrease, as a function of increasing stimulus coherence. Correspondingly, the model predicts the stop error rate to increase as the stimulus coherence increases (Figure 4D), with the effect present at almost the whole range of SSD tested in the experiment (Figure 4E). Additionally, and perhaps more surprisingly, the model predicts the SSRT to decrease as a function of increasing go stimulus coherence. Although SSRT is not an intrinsic parameter or entity in the optimal model, one can nevertheless estimate SSRT as one does from empirical data, by identifying the SSD at which approximately 50% stop accuracy is achieved. This last prediction is particularly intriguing for differentiating the race model and the optimal model, as the race model would not predict that go stimulus difficulty should influence the speed at which the stop signal is processed.

### 3.2. Human behavioral data

**Figure 5:**
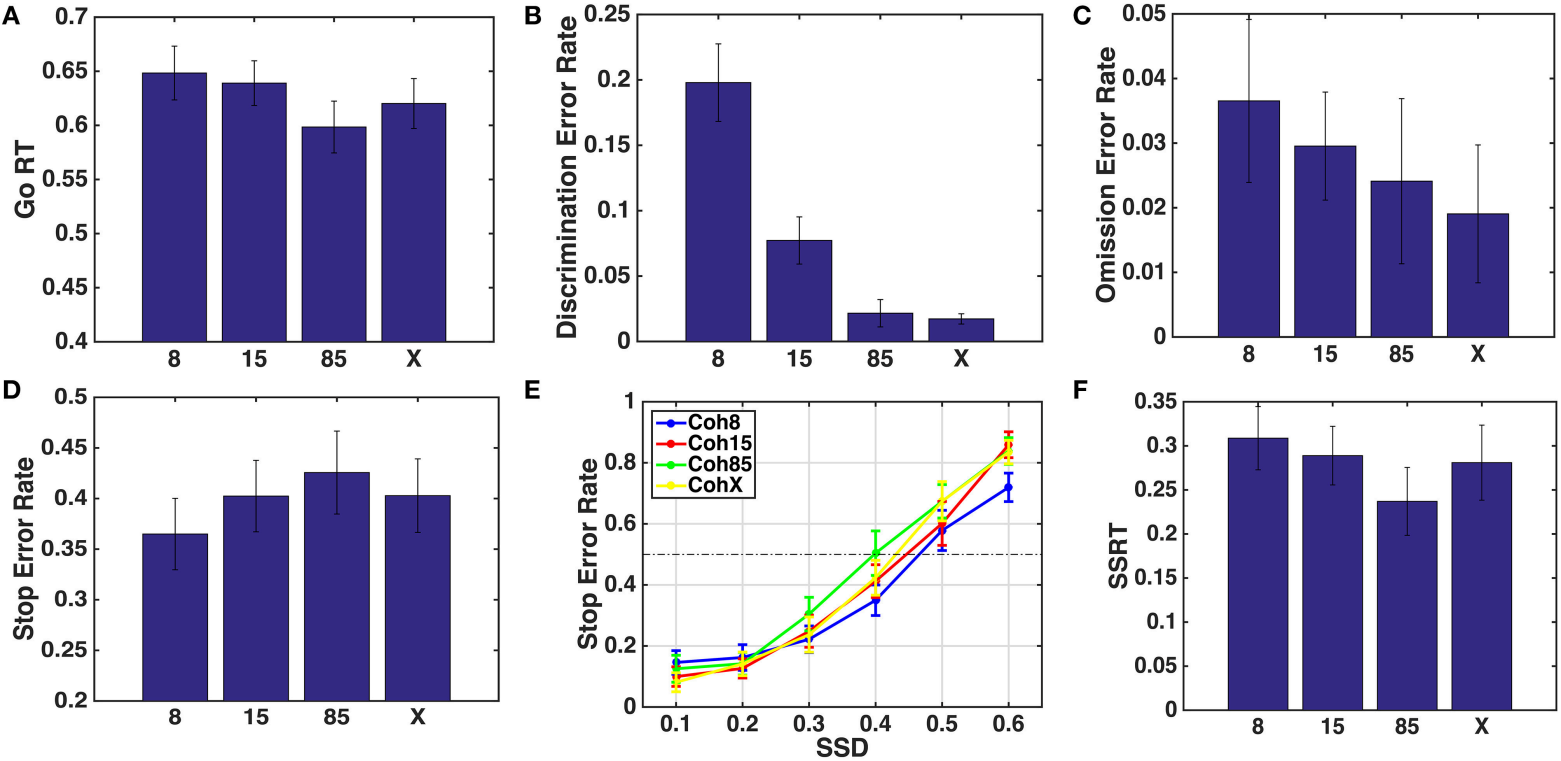
**Behavioral data for varying go stimulus discrimination difficulty**. 8, 15, 85 denote different coherences of random dot motion stimulus. X denotes square vs. circle discrimination task. **(A)** Mean Go RT decreased as the discrimination task became easier. Bar height indicates the mean of median Go RT (for each subject) for each condition. **(B)** Go discrimination error rate decreased as coherence increased. **(C)** Go omission error rate decreased with coherence. **(D)** Stop error rate increased with coherence. **(E)** Average inhibition function for different stimulus types. **(F)** SSRT decreased with coherence.

In this section, we show that the model predictions in Section 4 are confirmed by the human behavioral data, where 8, 15, and 85% denote different coherences of random dot motion stimulus, while “X” represents square vs. circle go stimulus. Figure 5 shows the behavioral results from the experiment. Figures 5A–C,F show that subjects' mean Go RT, discrimination error rate, omission error rate, and SSRT decreased with coherence, as predicted in Figure 4. Figure 5D shows that subjects' stop error rate increases with coherence. We used one-sided paired *t*-test to test the significance of differences in behavioral measures across different go stimulus difficulties, e.g., *H*_0_ : mean (Go RT for 8%) = mean (Go RT for 15%), *H*_1_ : mean(Go RT for 8%) < mean (Go RT for 15%). We also conducted the more conservative Wilcoxon rank test, which does not make the normality assumption that *t*-test makes, for completeness. As we detail in Supplementary Material, similar results are found using the two tests, except for the omission error, the trial type for which we have the least amount of data, since omission errors were rare. Here, we only discuss the results of the paired *t*-tests.

We found that mean Go RT significantly decreased as the coherence increased from 15 to 85% (*p* = 0.008, *t* = 2.63) and 8% to 85% (*p* = 0.012, *t* = 2.44), but not significant from 8% to 15% (*p* = 0.263, *t* = 0.64). In consideration of the long tail of the RT distribution (though this was ameliorated in the current study due to the response deadline), we computed the median Go RT of each subjects and then conducted paired *t*-test, which showed that median Go RT significantly decreased as coherence increased from 15 to 85% (*p* = 0.004, *t* = 2.87) and 8 to 85% (*p* = 0.002, *t* = 3.20), and showed a trend toward significance from 8 to 15% (*p* = 0.09, *t* = 1.35).

Paired *t*-tests for discrimination error rates were significant for all three cases, 8% to 15% (*p* < 10^−6^, *t* = 6.35), 15% to 85% (*p* = 0.002, *t* = 3.22), and 8% to 85% (*p* < 10^−6^, *t* = 6.65). The omission error rate only significantly decreased when the coherence increased from 8% to 85% (*p* = 0.01, *t* = 2.5), but not from 8% to 15% (*p* = 0.16, *t* = 1.00), not from 15% to 85% (*p* = 0.22, 0.77). Over all, the results suggest that Go RT, Discrimination Error and Omission Error decrease with coherence. In addition, the stop error rate also increased significantly when coherence increased from 8 to 85% (*p* = 0.03, *t* = −1.89), but not from 15% to 85% (*p* = 0.14, *t* = −1.10), and showed a trend toward significance from 8% to 15% (*p* = 0.06, *t* = −1.61). Altogether, the results suggest that stop error rate increases with coherence.

We used “smoothspline” function in Matlab to fit the inhibition function of each subject in each stimulus type and estimated the corresponding SSRT (Figure 5F) as the difference between median Go RT and the SSD at which 50% stop error rate is committed (Figure 5E). According to the paired *t*-test, SSRT significantly decreased as the coherence increased from 8 to 85% (*p* = 0.02, *t* = 2.41), but not from 8 to 15% (*p* = 0.23, *t* = 0.79), and showed a trend toward significance from 15 to 85% (*p* = 0.09, *t* = 1.40). Over all, the results suggest that SSRT decreases with coherence.

We also included a more classical circle vs. square go discrimination task, because stop-signal tasks typically use highly discriminable go stimuli such as circle vs. square. As we are among the first to use the random-dot coherent motion stimuli for the go task, as well as the first to systematically degrade the go stimulus in the stop-signal task, we wanted to make sure that the easiest condition (85% coherence) produces comparable data to the more commonly used shape discrimination task. Figure 5 shows that this is indeed the case across the behavioral measures we examined.

## 4. Discussion

In this work, we investigated the computational and behavioral consequences of manipulating stimulus discriminability in the stop-signal task. We simulated our previously proposed optimal decision-making model (Shenoy and Yu, 2011) to derive behavioral predictions, and presented novel experimental data that broadly validated these predictions. Interestingly, the SSRT, which is thought to reflect the stopping ability of the subject, is found to significantly decrease with increasing difficulty of the go stimulus discrimination task. This directly contradicts the independence assumption of the race model (Logan and Cowan, 1984), as well as its more complex variants that assume a simple inhibitory interaction between the go and stop processes that is independent of the go stimulus discriminability (Boucher et al., 2007). Together, our results imply that there exist intrinsic and complex interactions between *go* and *stop* processing, much as that postulated by our optimal decision-making model for stopping behavior (Shenoy and Yu, 2011). More generally, the broad concurrence between model predictions and behavioral data demonstrate the normative predictive power of the optimal model, as well as the specific model assumptions that humans readily internalize environmental statistics and adopt decision policies that are normative and context-sensitive.

The difference between the race model and the optimal decision-making model is not only that of complexity or the nature of interactions between the stop and go processes, but also that of levels of analysis, in the parlance of Marr (1982). The race model is primarily an algorithmic model, while the optimal model is primarily a computational model. That means for a more meaningful comparison, it would be worthwhile to consider an algorithmic description of the optimal model that is more directly comparable to the race model (or, alternatively, a computational description of the race model, which is harder to obtain). Such an analysis was done in a previous paper (Shenoy and Yu, 2011), which showed that while the model would not be able to *predict* changes in stopping behavior as a consequence of changes in the reward structure of the task, the parameters of a diffusion-model implementation of the race model (see Shenoy and Yu, 2011 for more details) can be fit to different experimental conditions in a *post hoc* manner in order to capture qualitative changes in behavior. Likewise, one could fit parameters of a diffusion model equivalent of the race model to “capture” behavioral changes as a function of go stimulus signal-to-noise ratio, but again, it would be a *post hoc* result, not a normative predictive process as the optimal decision-making model excels in.

In the current paper, the parameter *q*_*d*_, which specifies the noisiness of the sensory data related to the go stimulus, is left as a free parameter. While we kept *q*_*d*_ monotonically increasing as a function of increasing stimulus coherence (a rational choice), its values for different coherence conditions were somewhat arbitrarily chosen. Although the qualitative nature of the model predictions (changes in go RT, stop error rate, and SSRT as a function of go stimulus coherence) is relatively robust with respect to the precise choice of *q*_*d*_, an even better approach would be to fit *q*_*d*_ for each subject in a pure 2AFC task, identical to the stop-signal task except for the total absence of the stop signal, as 2AFC behavior is fairly well captured and understood as a variant of the sequential probability ratio test (Gold and Shadlen, 2002; Bogacz et al., 2006; Frazier and Yu, 2008; Dayanik and Yu, 2012; Shenoy and Yu, 2012), which can be parameterized by essentially the same *q*_*d*_ variable. In that case, *q*_*d*_ would then not be a free parameter in the stop-signal task but one derived from a separate 2AFC session for each subject, and we would then be able to see whether stopping behavior really follow quantitatively from the optimal inference and decision-making process, as predicted by the model.

Another important direction of future research is a better theoretical understanding of the algorithmic aspects of the optimal model, in particular what determines SSRT in this model. While SSRT is not intrinsic to the optimal model, as it is in the race model, it is nevertheless possible to “measure” SSRT for the optimal model based on simulated trial outcomes, just as is done empirically for human data. Related to this, it is unclear *why* SSRT should decrease in the optimal model for increasing coherence, which we showed to be both predicted by model simulations and exhibited by human data in this paper. Specifically, as the go task gets easier, subjects make more stop errors due to faster Go RT, even though SSRT decreases—it just does not decrease sufficiently to counter the faster go RT. Future work is needed to better understand the nature of SSRT in the context of the optimal model, as well as, in general, a better algorithmic understanding of the relationship between the optimal model and the race model.

Although this work was mostly focused on computational modeling and behavioral data analysis, it has implications for the neuroscientific study of inhibitory control as well. The race model has helped to advance the neuroscience of inhibitory control, by relating neural activities in various brain regions, such as the frontal eye field (Hanes et al., 1998) and superior colliculus (Pare and Hanes, 2003), to the go and stop processes. But as the race model does not address how different cognitive processes contribute to stopping behavior, it is also limited in its ability to anticipate or explain cognitive modulations of neural activities involved in inhibitory control. Given the ability of the computationally more sophisticated optimal model to explain a wider range of behavioral data, we can expect that it will also lead to novel and interesting interpretations of neural activation patterns related to inhibitory control, and perhaps guide future neuroscientific experimentation. Indeed, we have already used the optimal model to identify a brain region (dorsal anterior cingulate cortex) as having fMRI BOLD response consistent with encoding an unsigned prediction error (“Bayesian surprise”) related to the prior belief of whether the upcoming trial will be a stop or go trial (Ide et al., 2013), and shown that this prediction signal is altered in young adults at risk for developing stimulant addiction (Harlé et al., 2014), a condition known to be associated with impaired inhibitory control and specifically stopping behavior. Prior to this model-based fMRI study, it was thought that the anterior cingulate cortex was one of many areas generally involved in preparing or executing the “go” response. In the context of the optimal model, we now know that this area, unlike the other cortical areas, is specifically involved in reporting the surprise signal, which just happens to be greater on stop trial than go trial on average, because stop trials are generally rare. This provides just one example of how a statistically sophisticated model facilitates a richer and more theory-driven exposition of the neural basis of inhibitory control.

## Author contributions

NM implemented all the code related to the models and data analysis, generated most of the figures, and drafted the manuscript. AJY supervised the experimental design, data collection, model implementation, data analysis, and figure generation, as well as manuscript preparation.

## Funding

This work was in part funded by a NSF CRCNS grant (BCS-1309346) to AJY.

### Conflict of interest statement

The authors declare that the research was conducted in the absence of any commercial or financial relationships that could be construed as a potential conflict of interest.
