# Supplementary material for: Inseparability of Go and Stop in Inhibitory Control: Go Stimulus Discriminability Affects Stopping Behavior
Source: Front Neurosci. 2016 Mar 22;10:54. doi: 10.3389/fnins.2016.00054 (PMC4801867; doi:10.3389/fnins.2016.00054)
Supplement: Supplementary file 1 [file DataSheet1.PDF]

Supplementary Material: Inseparability of Go and Stop in Inhibitory Control: Go Stimulus Discriminability Affects Stopping Behavior

Ning Ma[1], **Angela J. Yu**[2,*]

*Correspondence:
Angela Yu
ajyu@ucsd.edu

1 WILCOXON SIGNED-RANK TEST

Based on the the Wilcoxon rank test, mean Go RT significantly decreased as the coherence increased from 8% to 15% ($p = 0.05$, $rank = 148$), from 15% to 85% ($p = 0.009$, $rank = 168$) and from 8% to 85% (($p = 0.014$, $rank = 164$)). Note that in consideration of the long tail of the RT distribution (though this was ameliorated in the current study due to the response deadline), we computed the median Go RT of each subjects and ran Wilcoxon test to see whether median Go significantly changes with stimulus perception difficulty. Again, we got similar test results. The Wilcoxon rank test were significant for all three cases. Median Go RT significantly decreased as coherence increased from 8% to 15% ($p = 0.03$, $rank = 155$), from 15% to 85% ($p = 0.006$, $rank = 172$), and from 8% to 15% ($p = 0.005$, $rank = 174$).

Wilcoxon rank test for discrimination error rate were significant for all three cases, 8% to 15% ($p < 10^{-4}$, $rank = 189$), 15% to 85% ($p = 0.001$, $rank = 187$), and 8% to 85% ($p < 10^{-4}$, $rank = 210$). The omission error rate significantly decreased when the coherence increased from 8% to 85% ($p = 0.01$, $rank = 139$) and from 15% to 85% ($p = 0.007$, $rank = 102$), but not from 8% to 15%($p = 0.11$, $rank = 114$). Stop error rate increased significantly when coherence increased from 8% to 85% ($p = 0.04$, $rank = 60$), but not from 15% to 85% ($p = 0.11$, $rank = 64$), and showed a trend towards significant from 8% to 15% ($p = 0.05$, $rank = 61$).

Wilcoxon test showed that SSRT significantly decreased as the coherence increased from 8% to 85% ($p < 0.04$, $rank = 31$), but not from 8% to 15% ($p = 0.27$, $rank = 23$), and showed a trend towards significant from 15% to 85% ($p = 0.10$, $rank = 34$).
